# Supplementary figures and images for: Loss of Flotillin-2 enhances trastuzumab emtansine internalization and cytotoxicity by relieving negative regulation of HER2 internalization in HER2-amplified cancers
Source: bioRxiv. 2026 May 19:2026.05.15.725439. Preprint. [Version 1] doi: 10.64898/2026.05.15.725439 (PMC13228603; doi:10.64898/2026.05.15.725439)

Figure S1

**A**

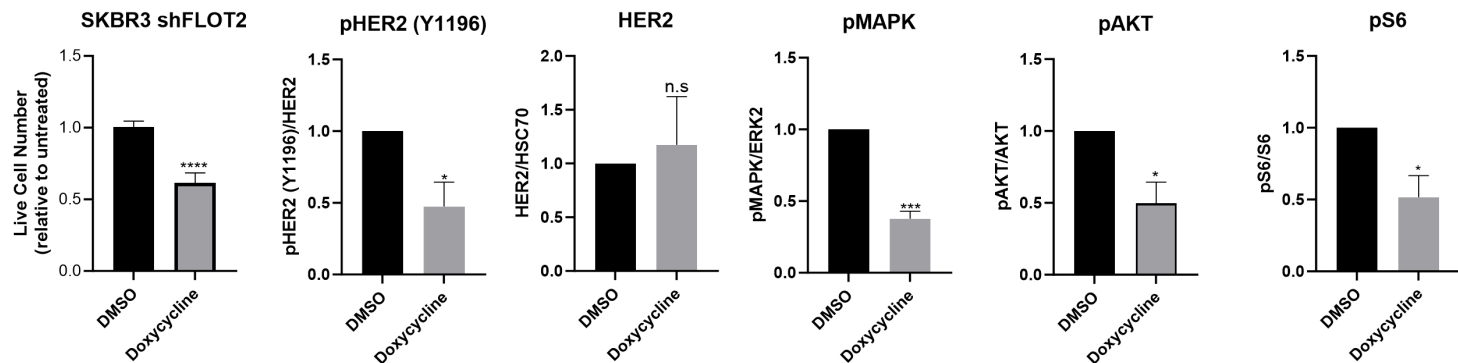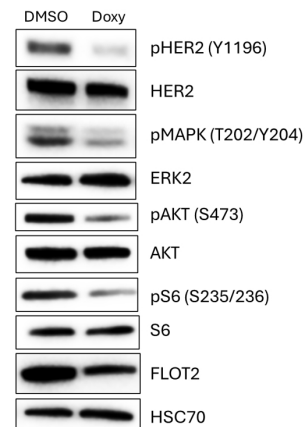

**B**

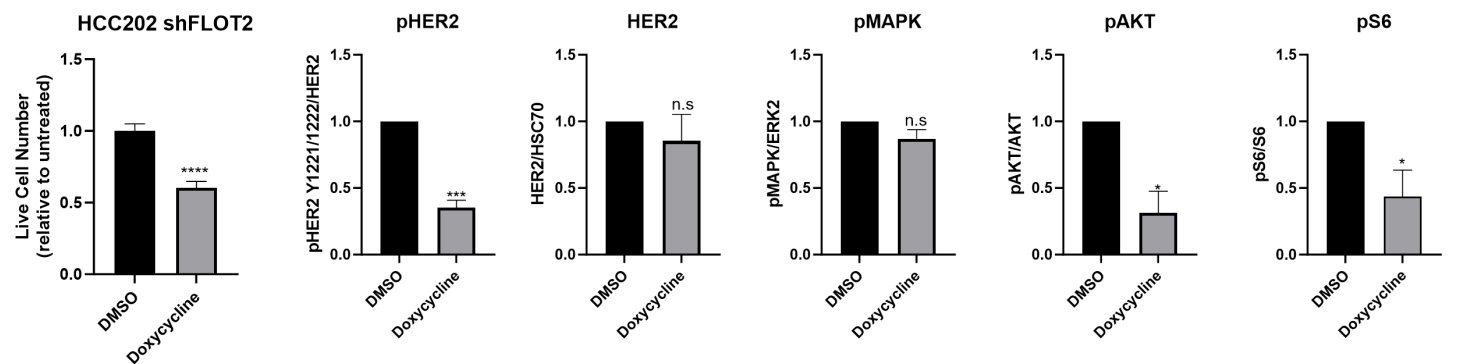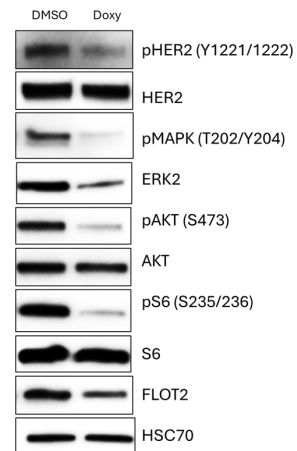

**C**

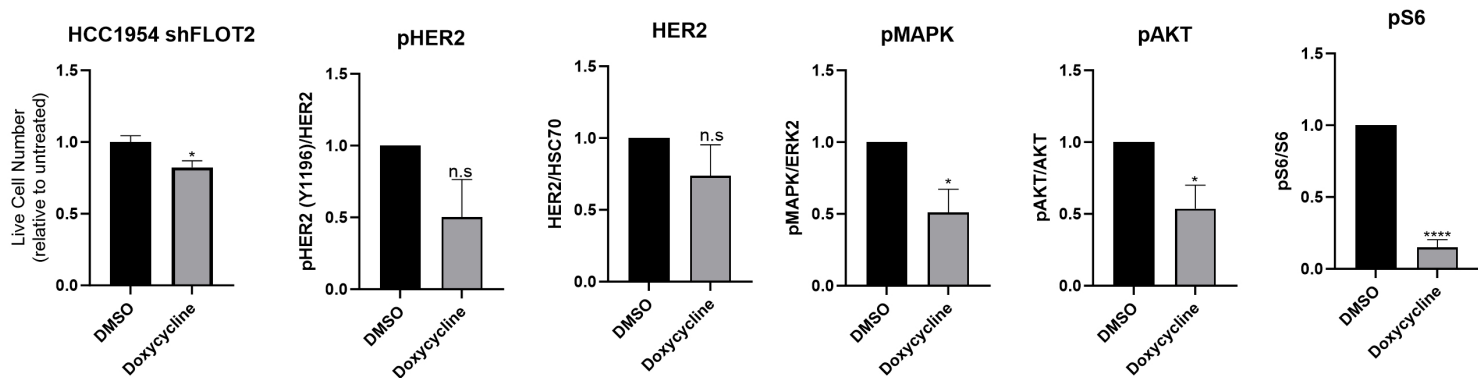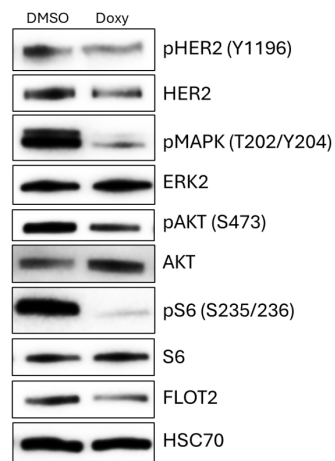

Supplement: Supplement 1 — A) SKBR3 shFLOT2 cells were treated +/− 500 ng/mL doxycycline for seven days prior to cell counting and five days prior to cell lysis. shFLOT2 lysates were probed for pHER2 (Y1196), HER2, pMAPK (T202/Y204), ERK2, pAKT (S473), AKT, pS6 (S235/236), S6, FLOT2, and HSC70 (loading control). Graphed data represent the average ±SEM of at least three independent experiments, and statistical analysis was performed by Student’s t-test. B) Same as A, with HCC202 shFLOT2 cells, and lysates were instead probed for pHER2 (Y1221/1222). Cells were treated with doxycycline for seven days prior to cell counting and cell lysis. C) Same as in A, with HCC1954 shFLOT2 cells. Cells were treated with doxycycline for seven days prior to cell counting and cell lysis. [file media-1.pdf]

Figure S2

**A**

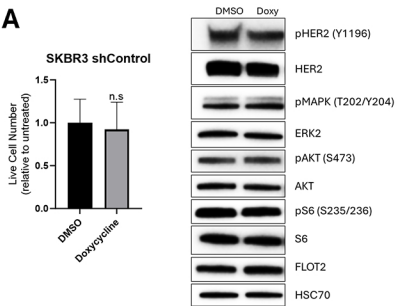

**B**

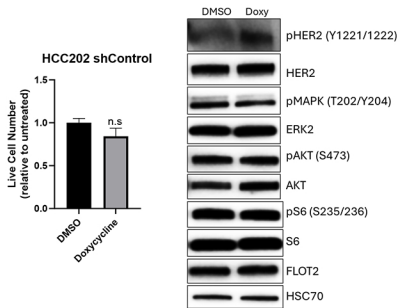

**C**

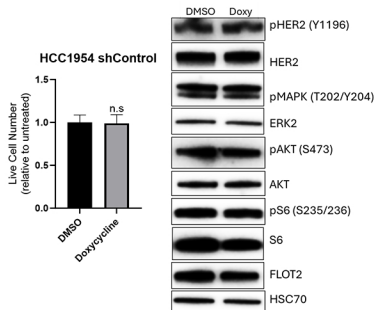

Supplement: Supplement 2 — A) SKBR3 shControl cells were treated +/− 500 ng/mL doxycycline for seven days prior to cell counting and five days prior to cell lysis. shControl lysates were probed for pHER2 (Y1196), HER2, pMAPK (T202/Y204), ERK2, pAKT (S473), AKT, pS6 (S235/236), S6, FLOT2, and HSC70 (loading control). Data represents the average ±SEM of at least three independent experiments, and statistical analysis was performed by Student’s t-test. B) Same as A, with HCC202 shControl cells, and lysates were instead probed for pHER2 (Y1221/1222). Cells were treated with doxycycline for seven days prior to cell counting and cell lysis. C) Same as in A, with HCC1954 shControl cells. Cells were treated with doxycycline for seven days prior to cell counting and cell lysis. [file media-2.pdf]

Figure S3

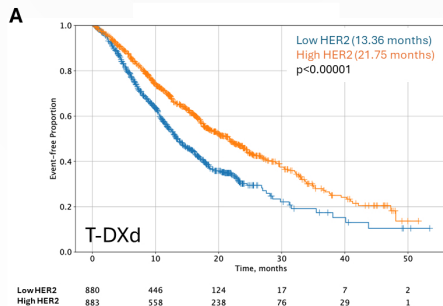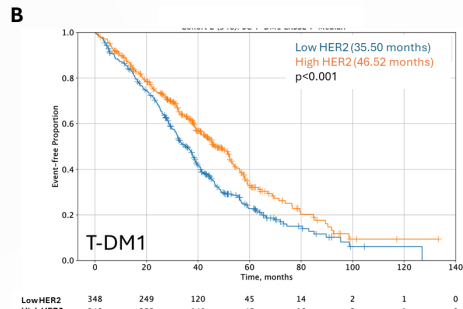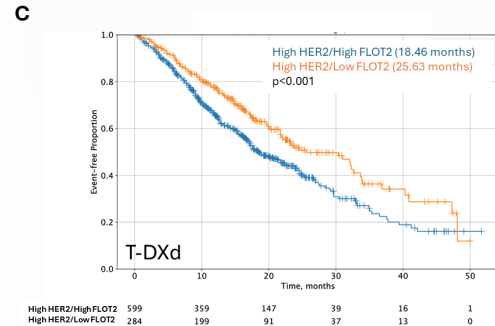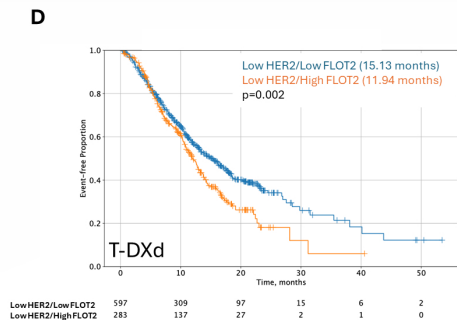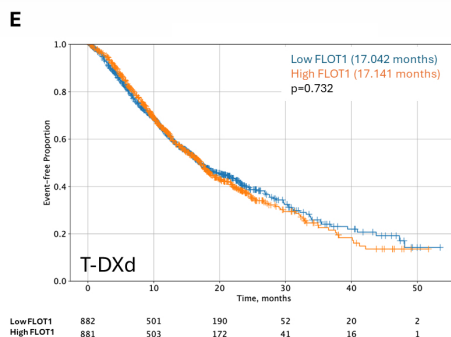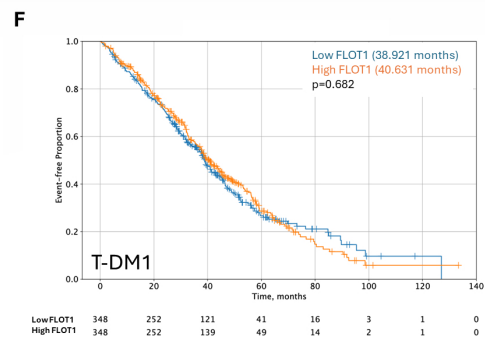

Supplement: Supplement 3 — A) Overall survival of patients in Caris dataset from T-DXd to last contact with high HER2 (orange; 21.747 months) vs low HER2 (blue; 13.357 months), p<0.00001. B) Overall survival of patients in Caris dataset from T-DM1 to last contact with high HER2 (orange; 46.521 months) vs low HER2 (blue; 35.499 months), p<0.001. C) Overall survival of patients in Caris dataset from T-DXd to last contact with high HER2/high FLOT2 (blue; 18.457 months) vs high HER2/low FLOT2 (orange; 25.629 months), p<0.001. D) Overall survival of patients in Caris dataset from T-DXd to last contact with low HER2/low FLOT2 (blue; 15.134 months) vs low HER2/high FLOT2 (orange; 11.943 months), p=0.002. E) Overall survival of patients in Caris dataset from T-DXd to last contact with high FLOT1 (orange; 17.141 months) vs low FLOT1 (blue; 17.042 months), p=0.732. F) Overall survival of patients in Caris dataset from T-DM1 to last contact with high FLOT1 (orange; 40.631 months) vs low FLOT1 (blue; 38.921 months), p=0.682. [file media-3.pdf]

Figure S4

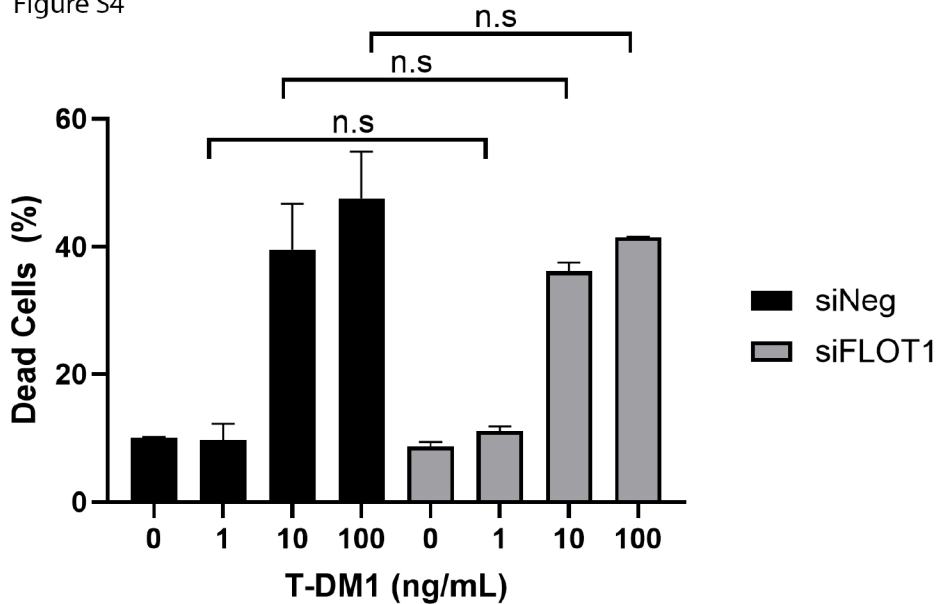

Supplement: Supplement 4 — SKBR3 cells were transfected with siNeg or siFLOT1 for 48 hours, replated, and then treated with 0, 1, 10 or 100 ng/mL T-DM1 in 1% FBS RPMI for 72 hours. Dead cell percentage was calculated using PI stain with the BioTek Cytation. Data represents the average ±SEM of at least three independent experiments, and statistical analysis was performed by Student’s t-test, comparing individual TDM1 concentration of siFLOT1 to siNeg. [file media-4.pdf]

Figure S5

**A**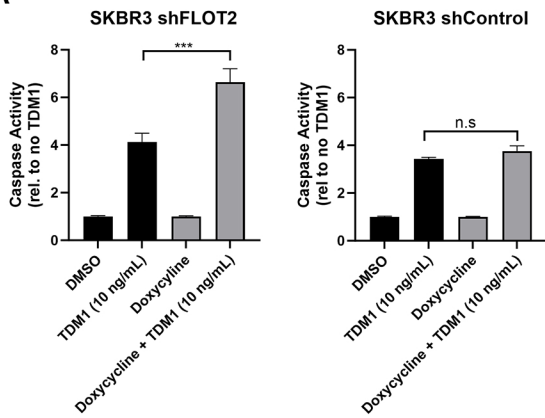**B**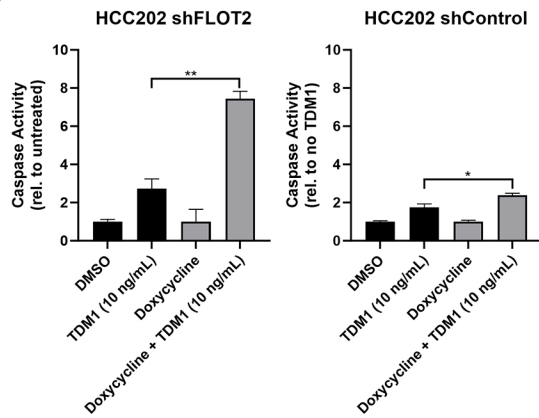**C**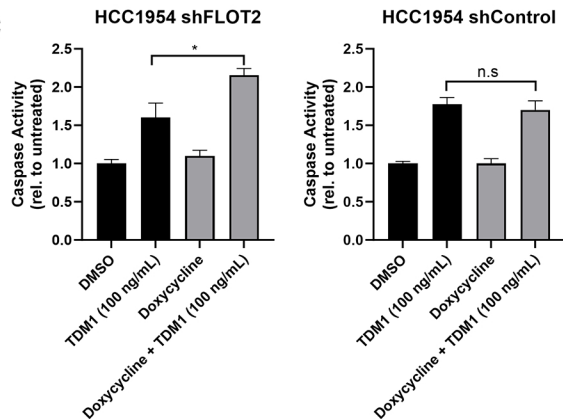

Supplement: Supplement 5 — A) SKBR3 shFLOT2 (left) or shControl (right) were treated +/− 500 ng/mL doxycycline for 48 hours and then treated with 10 ng/mL T-DM1 as indicated for 24 hours. Cells were then incubated with Caspase 3/7 reagent, luminescence was recorded, and values were normalized to DMSO control. Data represents the average ±SEM of at least three independent experiments, and statistical analysis was performed by Student’s t-test. B) Same as in A, with HCC202 shFLOT2 or shControl. C) Same as in A, with HCC1954 shFLOT2 or shControl, and 100 ng/mL T-DM1. [file media-5.pdf]

Figure S6

**A**

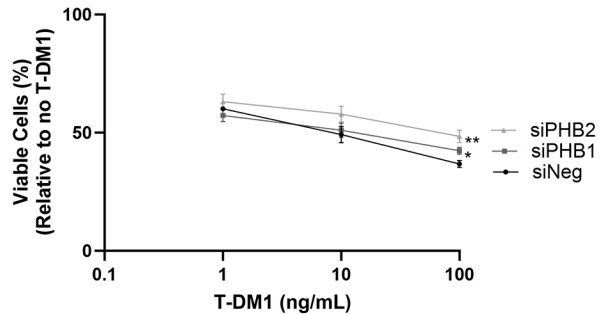

**B**

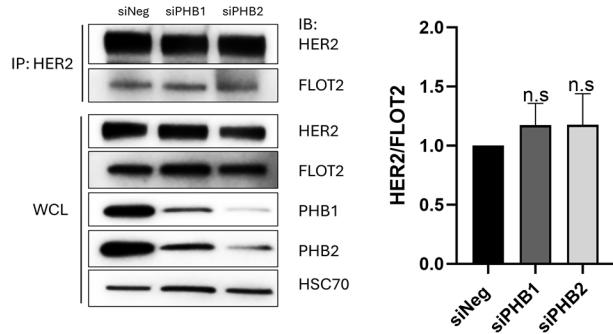

Supplement: Supplement 6 — A) SKBR3 cells were transfected with siNeg, siPHB1 or siPHB2 for 72 hours, replated, and treated with 0, 1, 10 or 100 ng/mL T-DM1 for 48 hours. Dead cell percentage was calculated using PI stain with the BioTek Cytation. Viable cell percentage was calculated by subtracting dead cell percentage from 100. These percentages were normalized to 0 ng/mL TDM1 for each transfection condition. Data represents the average ±SEM of at least three independent experiments, and statistical analysis was performed by Student’s t-test, comparing different T-DM1 concentrations in either siPHB1 or siPHB2 to siNeg T-DM1 concentrations. B) Transfected SKBR3 cells from A were lysed, immunoprecipitated for anti-HER2, and immunoblotted for HER2 and FLOT2. Whole cell lysate was immunoblotted for HER2, FLOT2, PHB1, PHB2 and HSC70 (loading control). Immunoprecipitation was quantified for HER2/FLOT2, and normalized to siNeg (right). Data represents the average ±SEM of at least three independent experiments, and statistical analysis was performed by Student’s t-test. [file media-6.pdf]
